# Different adhesion properties of highly and poorly metastatic HT-29 colon carcinoma cells with extracellular matrix components: role of integrin expression and cytoskeletal components

**DOI:** 10.1038/sj.bjc.6690614

**Published:** 1999-08

**Authors:** J Haier, M Nasralla, G L Nicolson

**Affiliations:** The Institute for Molecular Medicine, 15162 Triton Lane, Huntington Beach, CA 92649, USA

**Keywords:** colorectal cancer, metastasis, adhesion, cytoskeleton, ECM, integrins

## Abstract

Integrin-mediated tumour cell adhesion to extracellular matrix (ECM) components is an important step in the development of metastatic lesions. Thus, integrin expression and integrin-mediated adhesion of colon carcinoma cells to various ECM components was examined. Poorly (HT-29P) and highly (HT-29LMM) liver-metastatic colon carcinoma cells were used to study the rates of adhesion to collagen I (C I), collagen IV (C IV), laminin (LN), fibronectin (FN), or vitronectin (VN) in a static adhesion assay (10–120 min). Cells were untreated or treated with oligopeptides (RGD, GRGDS, YIGSR, RGES), anti-integrin antibodies, or colchicine, nocodazole, cycloheximide, acrylamide or cytochalasin D (to disrupt cytoskeletal structures). Both cell lines expressed similar patterns of integrin expression (α_2_, α_3_, α_6_, α_v_, β_1_, β_4_ and β_5_) by immunocytochemistry and immunoprecipitation. HT-29LMM cells showed significantly higher rates of adhesion to LN (*P* < 0.001) and FN (*P* < 0.001), but significantly poorer rates of adhesion to C I (*P* < 0.05) and C IV (*P* < 0.001) than HT-29P cells, respectively, adhesion to VN was insignificant. RGD and GRGDS inhibited HT-29LMM cell adhesion to FN only. Pretreatment with anti-β_1_ or anti-α_2_ integrin subunits suppressed adhesion to C I and C IV, and adhesion to LN was inhibited with anti-β_1_ or anti-α_6_ integrin. Anti-β_1_ or anti-α_v_ blocked adhesion to FN. Pretreatment of cells with cytochalasin D, cycloheximide or acrylamide inhibited adhesive interactions of both cell lines to the ECM components. In contrast, colchicine and nocodazole had no effect. The results demonstrate that adhesion of HT-29 cells to ECM is mediated, in part, by different integrins, depending on the substrate. Poorly and highly metastatic HT-29 cells possessed different patterns of adhesion to the various ECM substrates, but these differences were not due to different expression of integrin subunits. The results also suggested that the initial adhesion of poorly or highly metastatic HT-29 cells to ECM components requires, in part, the presence of native action and intermediate filaments, but not of microtubules. Thus the adhesion of tumour cells to ECM components may be dependent on signal transduction and assembly of microfilaments. © 1999 Cancer Research Campaign


*British Journal of Cancer* (1999) **80**(12), 1867–1874
© 1999 Cancer Research Campaign
Article no. bjoc.1999.0614

### Different adhesion properties of highly and poorly metastatic HT-29 colon carcinoma cells with extracellular matrix components: role of integrin expression and cytoskeletal components

**J Haier, M Nasralla and GL Nicolson**

The Institute for Molecular Medicine, 15162 Triton Lane, Huntington Beach, CA 92649, USA

**Summary** Integrin-mediated tumour cell adhesion to extracellular matrix (ECM) components is an important step in the development of metastatic lesions. Thus, integrin expression and integrin-mediated adhesion of colon carcinoma cells to various ECM components was examined. Poorly (HT-29P) and highly (HT-29LMM) liver-metastatic colon carcinoma cells were used to study the rates of adhesion to collagen I (C I), collagen IV (C IV), laminin (LN), fibronectin (FN), or vitronectin (VN) in a static adhesion assay (10–120 min). Cells were untreated or treated with oligopeptides (RGD, GRGDS, YIGSR, RGES), anti-integrin antibodies, or colchicine, nocodazole, cycloheximide, acrylamide or cytochalasin D (to disrupt cytoskeletal structures). Both cell lines expressed similar patterns of integrin expression ($\alpha_2$, $\alpha_3$, $\alpha_6$, $\alpha_v$, $\beta_1$, $\beta_4$ and $\beta_5$) by immunocytochemistry and immunoprecipitation. HT-29LMM cells showed significantly higher rates of adhesion to LN ($P < 0.001$) and FN ($P < 0.001$), but significantly poorer rates of adhesion to C I ($P < 0.05$) and C IV ($P < 0.001$) than HT-29P cells, respectively, adhesion to VN was insignificant. RGD and GRGDS inhibited HT-29LMM cell adhesion to FN only. Pretreatment with anti-$\beta_1$ or anti-$\alpha_2$ integrin subunits suppressed adhesion to C I and C IV, and adhesion to LN was inhibited with anti-$\beta_1$ or anti-$\alpha_6$ integrin. Anti-$\beta_1$ or anti-$\alpha_v$ blocked adhesion to FN. Pretreatment of cells with cytochalasin D, cycloheximide or acrylamide inhibited adhesive interactions of both cell lines to the ECM components. In contrast, colchicine and nocodazole had no effect. The results demonstrate that adhesion of HT-29 cells to ECM is mediated, in part, by different integrins, depending on the substrate. Poorly and highly metastatic HT-29 cells possessed different patterns of adhesion to the various ECM substrates, but these differences were not due to different expression of integrin subunits. The results also suggested that the initial adhesion of poorly or highly metastatic HT-29 cells to ECM components requires, in part, the presence of native action and intermediate filaments, but not of microtubules. Thus the adhesion of tumour cells to ECM components may be dependent on signal transduction and assembly of microfilaments.

**Keywords:** colorectal cancer; metastasis; adhesion; cytoskeleton; ECM; integrins

The location of secondary tumours is determined, in part, by anatomical blood flow and by interactions between tumour cells and host organs (Nicolson, 1988*a*). During hematogenous metastasis formation some tumour cells show selective colonization of various organs. This is the result of a multistep sequential process, determined by specific properties of the tumour cell as well as the host organ (Nicolson, 1988*a*). The adhesion of blood-borne malignant cells to microvascular vessel walls and the migration of the metastatic cells to the underlying extracellular matrix (ECM) and eventually into the host organ are very important in this process (Nicolson, 1989). During the adhesive interactions between tumour cells and the vascular ECM certain molecules function for primary ECM contact, adhesion stabilization and cell migration into the host organ (Doerr et al, 1989; Nicolson, 1989, 1991). In various organs ECM composition has been found to be different which could be important in tumour cell–ECM adhesion (Nicolson, 1995). Additionally, some tumours, such as colon carcinomas, produce their own ECM components, and these components can modify peritumoural ECM composition (Burtin et al, 1983; Grigioni et al, 1986). In some tumour systems the expression of ECM components by tumour cells, such as the expression of oncofetal fibronectin (FN) by colorectal carcinomas, correlates with poor prognosis (Inufusa et al, 1995). Also, various immunohistochemical studies have investigated the expression of ECM-binding integrins in normal colon tissue and in colon carcinomas of different stages, including their metastases (Lindmark et al, 1993; Takazawa, 1995). Although certain patterns of integrin expression were found, it was apparent that there is a broad spectrum of expression of these molecules (reviewed by Agrez and Bates, 1994). The reported differences in immunostaining patterns include changes in integrin subunit expression with clinical stage, and over- or underexpression of certain integrins have been observed in malignant colon tissues compared to normal epithelium (Stallmach et al, 1992; Streit et al, 1996).

Once tumour cell adhesion has occurred it must be stabilized, and integrins and integrin-mediated signal transduction are apparently important in this process. Transmembrane integrin receptors can be linked through the cell membrane to cytoskeleton components (Chen et al, 1994), such as microfilaments, and they are thought to be important factors in adhesion stabilization, but their

*Received 27 July 1998*
*Revised 16 February 1999*
*Accepted 17 February 1999*

*Correspondence to:* GL Nicolson roles in the initial events of adhesion are only partially understood. Cytoskeletal proteins can be linked to transmembrane receptors in adhesion plaques to form supramolecular complexes (reviewed by Schwarz et al, 1995). Important integrins in this process are the $\beta_1$-integrins, which are linked to actin, $\alpha$-actinin and talin, which are further connected to various other cytoskeleton proteins like vinculin and zyxin (Horwitz et al, 1986; Yamada and Geiger, 1997). Integrin signals that mediate interactions with cytoskeleton components can be induced by the binding and clustering of integrins at the cell surface (Jewell et al, 1995; Miyamoto et al, 1995). This process may also be linked to phosphorylation events, because during adhesion phosphorylation, mainly on tyrosine residues, occurs and this, in turn, may activate several intracellular signaling cascades (Kornberg et al, 1991; Akiyama et al, 1994). Different complexes may be involved, such as focal adhesion kinase-paxillin (Guan et al, 1991; Akiyama et al, 1994; Jewell et al, 1995) or Grb2-SOS (Mainiero et al, 1995) complexes. Recent studies have shown that cytoskeleton components are also linked to signal transduction processes (Ben-Ze'ev, 1997). These complex interactions form signalling cascades that are organized in hierarchic systems and connect integrin-mediated signalling with other intracellular signalling and adhesion systems (Mainiero et al, 1995).

Much of the data on integrin-mediated signal transduction were obtained using fibroblasts or non-epithelial tumour cells, and the differences in phosphorylation patterns and involvement of cytoskeleton components could be related to cell origin. Since cell signalling cascades are likely to be different in different cell systems, we used poorly and highly liver-metastatic human colon carcinoma cell lines of epithelial cell origin to examine the adhesion properties to various ECM components. We also examined the involvement of intermediate filaments, actin and microtubules in adhesive interactions of these cells with ECM components.

### MATERIALS AND METHODS

### Materials

Dulbecco's modified Eagle's medium/F12 medium (DMEM/F12, 1:1 v/v) were purchased from Irvine Scientific; fetal bovine serum (FBS) from GibcoBRL; FN, laminin (LN), collagen I (C I) and IV (C IV) were obtained from Collaborative Biomedical Products. Vitronectin (VN) was purified as described (Yatohgo et al, 1988), and its purity tested using Western blotting. Mouse anti-$\alpha_3$- and $\alpha_5$-integrins monoclonal antibodies (mAb) were from Calbiochem; anti-$\alpha_v$- and $\beta_3$-integrin from Zymed; anti-$\beta_1$-, $\alpha_1$-, $\alpha_2$- and $\alpha_6$-integrin, rat- and mouse-IgG, goat-anti-mouse- and goat-anti-rat-IgG-HRP were from Harlan Serotec. Anti-$\beta_4$-integrin was obtained from Chemicon, anti-$\beta_5$-integrin was from Upstate Biotech. All anti-integrin antibodies (except anti-$\beta_5$) had blocking properties for adhesion. RGD-, GRGDS-, RGES- and YIGSR-peptides were obtained from Sigma. Cytochalasin D and nocodazole were products of Calbiochem; acrylamide was obtained from Biorad; colchicine and cycloheximide from Sigma; and CalceinAM from Molecular Probes.

### Cell lines

We used poorly (HT-29P) and highly (HT-29LMM) liver-metastatic colon carcinoma cells to study adhesion properties. The HT-29LMM subclone was obtained from intraspleenic injection in nude mice (Price et al, 1989). Cells were cultured in DMEM/F12 medium containing 10% FBS without antibiotics in humidified 5% carbondioxide 95% air at 37°C. Only cells of fewer than 15 passages were used. Cells were harvested with Trypsin–EDTA during the log-phase of growth. After trypsinization, cells were resuspended in adhesion medium (DMEM/F12 1:1, bovine serum albumin (BSA) 1%) at room temperature for 30 min for reconstitution of surface proteins and washed extensively in calcium–magnesium-free phosphate-buffered solution (CMF-PBS).

### Adhesion assay

Microtiter plates (96-well, Corning) were coated with C I (50 μg ml$^{-1}$), C VI (50 μg ml$^{-1}$), LN (50 μg ml$^{-1}$), FN (50 μg ml$^{-1}$), VN (10 μg ml$^{-1}$) or 1% BSA (negative control) at 37°C for 3 h (150 μl per well). Effective concentrations were determined in previous experiments (data not shown). Blocking of non-specific binding sites was performed using 1% BSA (37°C, 30 min, 200 μl per well). During reconstitution of surface proteins in adhesion medium, cells were fluorescence-labelled with CalceinAM (15 μg per 15 ml). After washing, cells were resuspended in adhesion medium at a final concentration of $0.5 \times 10^6$ cells per ml. Control experiments were performed to demonstrate that the CalceinAM did not affect cell viability or adhesion properties.

Adhesion experiments were performed with 150 μl cell suspension per well for different times (10–120 min) as previously described (Haier et al, 1998). For investigation of integrin-mediated cell adhesion, cells were pretreated with monoclonal antibodies (mAb) to integrin subunits (with IgG as a negative control). Inhibition was calculated by comparing adhesion of untreated (set to 100% adhesion) and treated cells. All data are given as relative percentage of adhesion compared to untreated cells. In other experiments cells were incubated with colchicine (10–100 μgM, 15 min) or nocodazole (0.5–5 μg ml$^{-1}$, 60 min) to disrupt microtubules, or with cycloheximide (10–100 μM, 60 min) or acrylamide (10–100 μM, 60 min) to disrupt intermediate filaments or with cytochalasin D (5–50 μM, 15 min) to disrupt actin filaments prior to the adhesion assay. Pretreatment was done at 37°C. Experiments were performed in triplicate and repeated twice. In the case of cytochalasin D and nocodazole control cells were treated with the same amount of dimethyl sulphoxide (DMSO) (5 μl ml$^{-1}$) to exclude non-specific effects of the reconstitution solution. Control experiments were performed to document the effects of the drugs on disrupting cytoskeletal components in HT-29 cells.

### Integrin expression

Integrin expression was examined using cells bound to coated glass slides. Chamber slides (4-well, Nunc) were pretreated with ECM components or anti-integrin mAb (1 μg per 0.5 ml) using the same conditions as for microtiter-plates. Coating with non-specific mouse IgG was used as a negative control. Reconstituted untreated cell suspensions (0.5 ml per chamber) were added to ECM pretreated slides. Adhesion was performed as described above in FBS-free adhesion medium. After 120 min, non-adherent cells were removed by washing with CMF-PBS. Adherent cells were visualized with haematoxylin stain. Semiquantitative analysis was done using a Zeiss microscope. Additionally, integrins were immunostained at the cell surface. After incubation of cell

 *© 1999 Cancer Research Campaign*

**Table 1** Immunocytochemical staining of integrin subunits on HT-29 cells and binding of cells to immobilized anti-integrin mAbs

| Integrin | Expression | | Integrin | Cell binding | |
|---|---|---|---|---|---|
| | HT-29P | HT-29LMM | | HT-29P | HT-29LMM |
| Mouse IgG | – | – | Mouse IgG | – | – |
| Alpha 1 | – | – | Alpha 1 | – | – |
| Alpha 2 | +++ | +++ | Alpha 2 | +++ | +++ |
| Alpha 3 | +++ | +++ | Alpha 3 | +++ | +++ |
| Alpha 5 | – | – | Alpha 5 | – | – |
| Alpha 6 | ++ | ++ | Alpha 6 | +++ | +++ |
| Alpha v | + | + | Alpha v | + | + |
| Beta 1 | +++ | +++ | Beta 1 | +++ | +++ |
| Beta 3 | – | – | Beta 3 | – | – |
| Beta 4 | +++ | +++ | Beta 4 | +++ | +++ |
| Beta 5 | + | + | Beta 5 | + | + |

Cells were stained on glass slides or binding was measured to immobilized anti-integrin mAbs on glass slides according to Methods. Staining or binding: +++ intense; ++ moderate; + weak or single cell; – none.

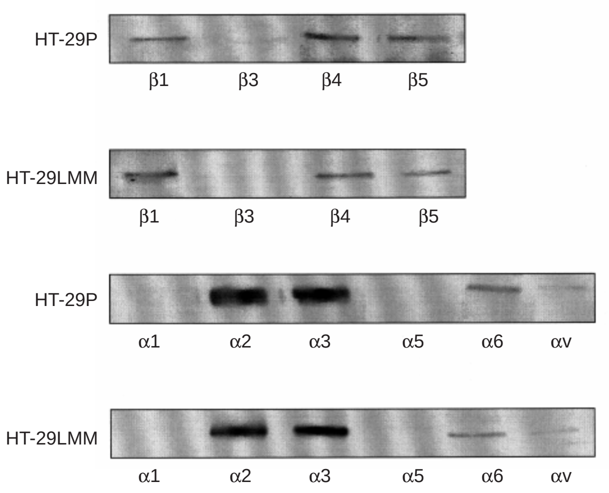

**Figure 1** Integrin expression on HT-29 cells. Immunoprecipitation of biotinylated surface proteins was performed using mAb against various integrin subunits. Precipitates were separated by SDS-PAGE under reduced conditions. Detection of (**A**) β-integrins and (**B**) α-integrins in HT-29P cells; (**C**) β-integrins and (**D**) α-integrins in HT-29LMM cells was done by streptavidin–alkaline phosphatase suspensions on ECM-coated slides, non-adherent cells were removed and the remaining cells were fixed using Conroy's solution (30% methanol, 70% acetic acid). After washing and blocking of non-specific binding (20% horse serum in PBS), cells were incubated with anti-integrin mAb (1 μg per 0.3 ml). Non-specific IgG was used as a negative control to evaluate background binding. For all experiments, non-specific staining was not seen. Following intensive washing, anti-species-specific mAb labelled with horseradish-peroxidase (1 μg per 0.3 ml) was added. Specific binding was visualized with AEC substrate (Coulter Immunotech) according to the manufacturer's guidelines. Counterstaining was performed with haematoxylin and semiquantitative analysis was performed by microscopy using a Zeiss microscope.

### Immunoprecipitation of biotinylated integrins

Cells were grown to 70–80% confluence in a 100-mm culture dish, culture medium was removed and cells were washed extensively with cold Buffer A (20 mM sodium hydrogen carbonate, 0.15 M sodium chloride (NaCl), pH 8.0). For biotinylation of surface molecules 1 mg NHS-LC-Biotin (Pierce) was added in 4 ml Buffer A (4°C for 90 min). After extensive washing with Buffer A, cells (~$10^7$ cells per dish) were lysed in 4 ml modified RIPA buffer (PBS containing 1.0% NP40, 0.25% deoxycholate, 0.1 mM phenylmethylsulphonyl fluoride, 0.1 mM TLCK, 0.1 mM iodoacetamide, 10 μg $ml^{-1}$ leupeptin and 10 μg $ml^{-1}$ aprotinin; pH 7.4) at 4°C for 30 min. Cell lysates were centrifuged at 13 000 ***g*** for 10 min to remove insoluble material. After preclearing each cell lysate (30 min, 37°C) with 20 μl anti-mouse-IgG-Agarose per ml, supernatants were incubated with 1–5 μg anti-integrin-mAb for 2 h (at 37°C or at 4°C overnight). Precipitates were obtained by addition of 20 μl anti-mouse (rat)-IgG-Agarose (2 h at 37°C or at 4°C overnight). The agarose beads were washed 5 × with 3 ml RIPA buffer, sodium dodecyl sulphate (SDS) sample buffer (100 μl) was added to beads and the mixtures were incubated at 95°C for 5 min. SDS polyacrylamide gel electrophoresis (SDS-PAGE) (10%) was performed under non-reduced or reduced conditions. After blotting to polyvinylidene difluoride (PVDF) membranes (100 V, 1 h), the membranes were blocked in blocking buffer (10 mM TRIS,50 mM NaCl, 1% Tween-20, pH 7.5) containing 3% BSA and incubated in blocking buffer (0.5% BSA) with streptavidin-AP (Boehringer-Mannheim) for detection of biotinylated integrins. After washing, the colour reaction was developed with BCIP/NBT solution (Zymed) and stopped with $dH_2O$.

### RESULTS

### Surface expression of integrins

The expression of different integrin subunits was evaluated using immunocytochemical staining of immobilized HT-29 cells. Intense staining was obtained for $\alpha_2$, $\alpha_3$, $\alpha_6$, $\beta_1$ and $\beta_4$ subunits. A weaker signal was seen using $\alpha_v$- and $\beta_5$-mAb. Integrin subunits $\alpha_1$, $\alpha_5$ and $\beta_3$ were not detected. Cells bound to immobilized mAb on glass slides in a relationship similar to that found by immunocytochemical localization of integrin receptors on immobilized cells. Lower amounts of cells bound to immobilized anti-$\alpha_v$ mAb than to the other anti-integrin-mAbs. Differences were not found between poorly (HT-29P) and highly (HT-29LMM) metastatic cell lines (Table 1).

### Immunoprecipitation of integrins

To confirm the immunocytochemical data and exclude that these results reflect changes in the accessibility of integrins but not in integrin surface expression, integrin subunits were evaluated using immunoprecipitation of biotinylated surface proteins. Intense bands were obtained for $\alpha_2$, $\alpha_3$, $\alpha_6$, $\beta_1$ and $\beta_4$ subunits. Weaker bands were seen using $\alpha_v$- and $\beta_5$-mAbs. Integrin subunits $\alpha_1$, $\alpha_5$ and $\beta_3$ were not detected. Differences were not found between poorly (HT-29P) and highly (HT-29LMM) metastatic cell lines (Figure 1). These results supported the findings that both cell lines expressed similar patterns of integrins on their surfaces.

*© 1999 Cancer Research Campaign*

**Table 2** Relative adhesion of poorly and highly metastatic HT-29 cells to various ECM components

| ECM component | HT-29P (Per cent adhesion) | | | HT-29LMM (Per cent adhesion) | | |
|---|---|---|---|---|---|---|
| | 10 min | 30 min | 120 min | 10 min | 30 min | 120 min |
| C I | 13 ± 5 | 51 ± 7 | 63 ± 3 | 2 ± 0 | 41 ± 3 | 58 ± 3[a] |
| C IV | 3 ± 1 | 27 ± 6 | 41 ± 9 | 0 ± 0 | 14 ± 3 | 19 ± 3[b] |
| VN | 0 ± 0 | 0 ± 0 | 1 ± 0 | 0 ± 0 | 0 ± 0 | 2 ± 0 |
| LN | 0 ± 0 | 7 ± 1 | 36 ± 2 | 2 ± 0 | 17 ± 1 | 45 ± 4[b] |
| FN | 0 ± 0 | 0 ± 0 | 1 ± 0 | 0 ± 0 | 2 ± 0 | 20 ± 8[b] |

Adhesion is expressed as % cell adhesion or relative fluorescence at different times compared to total fluorescence in the cell suspension; background fluorescence was calculated using adhesion to BSA-coated surface and was subtracted; background fluorescence was below 1% in all experiments. [a]$P < 0.05$; [b]$P < 0.001$ compared to HT-29P cells after 120 min.

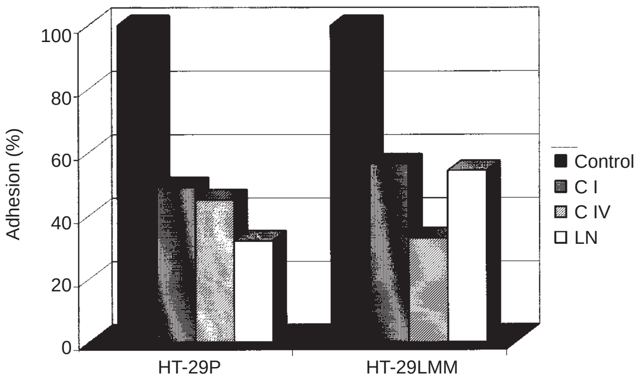

**Figure 2** Inhibition of adhesion of HT-29 cells to ECM components with cycloheximide. Cells were pretreated with 50 μM cycloheximide for 60 min at 37°C; relative amounts of adhered treated cells were compared to untreated cells (= 100% adhesion) after 120 min

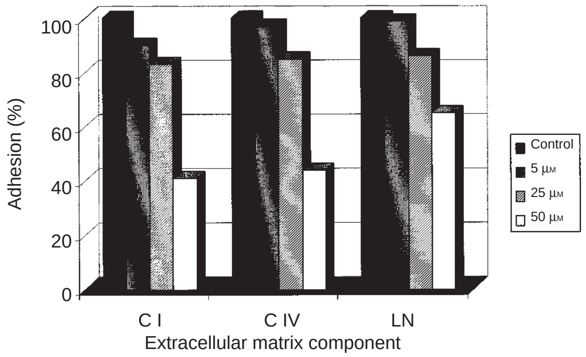

**Figure 3** Concentration-dependent inhibition of adhesion of HT-29LMM cells to ECM components. Cells were pretreated with 5–50 μM acrylamide for 60 min at 37°C, and relative amounts of adhered treated cells and untreated cells (= 100% adhesion) were compared after 120 min

### Adhesion to ECM components

Time-dependent cell adhesion to ECM components was measured until a plateau rate was obtained. Plateau values were obtained after 60 min using C I and C IV, whereas cells reached maximal adhesion to LN and FN after 90–120 min. In both cell lines high adhesion rates were observed to C I, C IV and LN; however, a poor rate of adhesion was demonstrated for FN using HT-29LMM cells, and FN-adhesion was not found using HT-29P cells. Adhesion to VN was not significant. HT-29LMM cells had significantly poorer rates of adhesion to C I ($P < 0.05$) and C IV ($P < 0.001$), but significantly higher rates of adhesion to LN ($P < 0.001$) and FN ($P < 0.001$) than HT-29P cells (Table 2).

### Inhibition of cell adhesion with anti-integrin antibodies

For tumour cell adhesion inhibition studies, values were compared after 120 min (plateau values). Results are given as relative amounts of attached treated cells compared to untreated cells (set to 100%). Pretreatment with anti-$\beta_1$ integrin subunit suppressed adhesion of both cell lines (HT-29P/HT-29LMM) to C I (7%/18%), C IV (13%/24%) and LN (0%/0%). Adhesion to C I and C IV was inhibited with anti-$\alpha_2$ (C I: 31%/40%; C IV: 66%/57%). Inhibition of adhesion to LN was achieved using anti-$\alpha_6$ (24%/16%). Anti-$\alpha_v$ blocked adhesion (55%) of HT-29LMM cells to FN. Negative controls with non-specific IgG showed no effect on cell adhesion. Cell adhesion was inhibited in a concentration-dependent manner (data not shown). Differences in adhesion were not seen using antibodies against $\alpha_1$, $\alpha_3$, $\alpha_5$, $\beta_3$ or $\beta_4$-subunits. The anti-$\beta_5$-integrin antibody had no blocking capabilities. Therefore, the potential role of $\beta_5$-integrins in integrin-mediated adhesion was not investigated.

**Table 3** Inhibition of HT-29 tumour cell adhesion to ECM components with cytoskeletal disrupting agents

| ECM component | Percent adhesion | | | | |
|---|---|---|---|---|---|
| | Cytochalasin D (10 μM) | Colchicine (50 μM) | Nocodazole (2.5 μg ml$^{-1}$) | Acrylamide (50 μM) | Cycloheximide (50 μM) |
| HT-29P | | | | | |
| C I | 1[a] | 96 | 99 | 27[b] | 49 |
| C IV | 3[a] | 96 | 106 | 25[b] | 45[b] |
| LN | 6[a] | 107 | 175 | 56[b] | 30[b] |
| HT-29LMM | | | | | |
| C I | 1[a] | 94 | 124 | 41[b] | 56[b] |
| C IV | 12[a] | 90 | 81 | 44[b] | 33[b] |
| LN | 11[a] | 118 | 88 | 65[b] | 54[b] |

Inhibitory effects are expressed as relative (%) adhesion compared to untreated cells (100%); effect of cytochalasin D and nocodazole were evaluated by comparison with DMSO-treated cells (5 μl ml$^{-1}$). [a]$P < 0.005$; [b]$P < 0.001$ compared to untreated cells.

 *© 1999 Cancer Research Campaign*

### Involvement of cell cytoskeleton in tumour cell adhesion

Treatment with colchicine did not affect tumour cell adhesion to ECM components (Table 3). Inhibitory effects are shown as per cent of attached treated cells compared with untreated cells (set to 100%). However, there was a concentration-dependent inhibition of adhesion using cycloheximide (30–56% adhesion) or acrylamide (44–66% adhesion) (Figures 2 and 3). Longer incubation times produced no further inhibitory effects, but the total adhesion was reduced. Additionally, we found strong inhibition (1–12% adhesion) using cytochalasin D, a drug that disrupts actin-filaments. Treatment with DMSO alone had only a minimal inhibitory effect. In previous studies it was shown that the concentrations of drugs that were used resulted in disruption of microfilaments and cytoskeleton components (Chopra et al, 1991; Seufferlein and Rozengurt, 1994; Tang et al, 1994), and this was confirmed in the present studies (data not shown). Significant inhibition of adhesion was demonstrated independent of the type of ECM component used as a substrate with either cell line after 30–120 min.

### DISCUSSION

Specific integrin-mediated interactions between tumour cells and ECM in particular host organs are thought to be important for organ-specific metastasis formation. In colon carcinomas integrin expression differs depending on the metastatic potential of the tumour cell. Additionally, the quantitative expression of ECM components is organ-dependent (Nicolson, 1988*b*), and relationships between ECM interaction and differentiation state or behaviour of colon carcinoma cells have been described (Grigioni et al, 1986; Boyd et al, 1988; Danecker et al, 1989). In our study, the properties of poorly or highly metastatic colon carcinoma cell lines were analysed during adhesion to different ECM components. Both colorectal cell lines expressed similar patterns of integrins. Differences were not seen in conventional immunostaining or in the binding of cells to immobilized antibodies against various integrins. Intense staining was found for $\alpha_2$-, $\alpha_3$-, $\alpha_6$- and $\beta_1$-integrin subunits in both cell lines. A weaker signal was seen using $\alpha_v$-mAb in both cell lines. Integrin subunits $\alpha_1$, $\alpha_5$ and $\beta_3$ were not detected. Cells bound to immobilized mAbs on glass slides similar to immunocytochemical localization of receptors on immobilized cells. Lower amounts of cells adhered to anti-$\alpha_v$ mAb than to the other positive integrins ($\alpha_2$, $\alpha_3$, $\alpha_6$, $\beta_1$). These results were confirmed using immunoprecipitation of biotinylated integrins. Taken together it was shown that the surface expression of the tested integrin subunits was comparable in both cell lines. Furthermore, the results suggested that adhesion of HT-29 cells is mediated, in part, by different integrins, depending on the ECM components used in the adhesion experiments.

Poorly and highly metastatic HT-29 colorectal carcinoma cells possessed different patterns of adhesion to the various ECM substrates. The main difference found was in the adhesion to FN, which was only seen with HT-29LMM cells. Using other subclones of HT-29 cells, Danecker et al (1989) found similar differences in the adhesion behaviour of colorectal carcinoma cells, with poorly differentiated cell clones adhering better to FN. In contrast to other studies (Schreiner et al, 1991; Lehmann et al, 1994; Herzberg et al, 1996), we detected little or no adhesion of HT-29P cells to FN or VN under the conditions used for the adhesion assays. In previous studies with the HT-29LMM subclone, a higher rate of adhesion to liver endothelial cells was demonstrated compared to endothelial cells derived from lung (Sawada et al, 1996). According to our inhibition data, binding to C I, C IV, LN and FN were mediated by different types of $\beta_1$-integrins. The integrin $\alpha_2\beta_1$ was identified as likely to be responsible for the adhesive interactions with the collagens, and the $\alpha_6$ $\beta_1$-integrin was likely to be involved in the binding to LN. As previously described (Haier et al, 1998), RGD-inhibition of adhesion to FN was probably due to interference with FN-$\alpha_v\beta_1$ integrin interactions.

In our study different adhesion properties of HT-29P and HT-29LMM cells were not caused by differences in integrin expression, because we could not demonstrate a correlation between integrin expression and adhesion to ECM components. Previously it has been shown that the integrin expression of HT-29 cells was also independent from the state of differentiation of these cells (Schreiner et al, 1991). The moderate adhesion to LN was not mediated by the $\alpha_6\beta_4$-integrin. These results are in contrast to other studies, where this integrin was responsible for adhesion to LN (Orian-Rousseau et al, 1998). This difference may explain that the adhesion to LN was only moderate whereas other groups found higher rates of adhesion to this ECM component (Herzberg et al, 1996; Orian-Rousseau et al, 1998). We cannot completely rule out that different $\beta_5$- or $\beta_6$-integrin expression is related to different adhesive properties to FN. Recently, the $\alpha_v\beta_6$ was found as a possible FN receptor on HT-29 cells, despite the very low levels of $\alpha_v\beta_6$ and the much higher levels of $\alpha_v\beta_5$ (Kemperman et al, 1997). Both β-subunits, which were associated with the $\alpha_v$ -subunit, were shown to be involved in adhesion of HT-29 cells to FN and VN (Lehmann et al, 1994). Various studies demonstrated that the $\alpha_5$-integrin might be involved in the tumorigenicity and metastatic behaviour of colorectal carcinoma cells. Using immunohistochemistry it was shown that less differentiated and metastatic lesions possessed higher expression of the $\alpha_5$-subunit than normal mucosa or primary well-differentiated tumours (Streit et al, 1996). Differences were also found at the mRNA level, where highly metastatic colon carcinoma cell lines had higher amounts of $\alpha_5$-mRNA than poorly invasive sublines. Transfection of poorly metastatic cells leads to an increase in tumorigenicity in athymic mice (Gong et al, 1997). However, these integrins are not involved in adhesion to collagens or LN, and differences in the expression would not explain different adhesive properties to these ECM components.

Our findings suggest that different intracellular events during cell adhesion may explain the differences in adhesion between poorly and highly metastatic colorectal tumour cells, or non-integrins mediated the differences in cell adhesion. Thus, overexpression of a single or few adhesion molecules alone cannot explain the different metastatic adhesive phenotypes. It is more likely that alterations in the affinity of integrins and their membrane distributions might be more important in the progression of colon carcinoma cells (Kim et al, 1994). Alternatively, differences in their metastatic behaviour may be explained by other tumour cell properties. The functional status of integrins, which are known for their activation or changes in affinity after binding to ECM components, is regulated by complex interactions with a number of cytosolic, cytoskeletal and membrane-bound proteins. Cells can modulate integrin-binding affinities and kinetics of interactions between integrin receptors and their adhesive ligands. These adhesion complexes serve both as recipients and as generators of signalling information that includes structural and signalling functions. For example, cell adhesion receptors can generate regulatory signals

© 1999 Cancer Research Campaign 

within cells that allow them to control cell adhesion, migration and invasion into the host organ.

Local regulation of cytoplasmic processes, including alteration of the cytoskeleton, secretion and the control of adhesion itself, are prominent features of integrin-mediated signalling (Gumbiner, 1996). As cells adhere to ECM components, integrins become localized in areas of focal contacts (Kornberg et al, 1991; Seftor et al, 1992). Focal contacts may function in signal transduction events, and they might be essential for some functions of $\beta_1$-integrins. Redistributions of integrins in forming focal contacts during adhesion can lead to activation of the intracellular integrin domain, modulating interactions with the cytoskeleton. Components of the tumour cell cytoskeleton (microtubules, microfilaments and intermediate filaments) have been reported in such complexes, and their organization can affect metastatic ability, since disruption of these components can result in decreases in metastatic properties. For example, B16 melanoma cells formed fewer lung colonies than control cells after treatment with cytochalasin D and cycloheximide. However, colchicine failed to inhibit lung colony formation (Ben-Ze'ev and Raz, 1985; Chopra et al, 1990). Neither colchicine nor cycloheximide treatment altered initial pulmonary arrest or adhesion to murine microvessel-derived endothelial cells (Chopra et al, 1988). The ability of tumour cells to aggregate homologous platelets is inhibited by the disruption of microfilaments and intermediate filaments but not on disruption of microtubules (Chopra et al, 1990). Mooney and colleagues (Mooney et al, 1995) found a rapid increase of F-actin in hepatocytes after spreading on different ECM components, but microtubules assembled slowly. In contrast, cell spreading could be completely inhibited by combining suboptimal doses of cytochalasin D and nocodazole, suggesting that intact microtubules can stabilize cells when the microfilament network is partially compromised.

In our study, we demonstrated that adhesion of colon carcinoma cells to ECM components can be inhibited by treatment with cycloheximide/acrylamide and cytochalasin D. However, no effect was found after disruption of microtubules with colchicine. Short incubation times with cycloheximide and similar results using acrylamide ruled out that the demonstrated effects were dependent on protein synthesis. Longer incubation times (up to 3 h) had no additional effects on inhibition, but the total amounts of adhesion were reduced. We conclude that the initial adhesion of poorly and highly metastatic HT-29 cells to ECM components requires, in part, presence of native intermediate filaments and actin filaments, but not microtubules. The results also suggest that adhesion of tumour cells to ECM components may be dependent on signal transduction, and native microfilaments appear to be necessary for this process.

The cytoskeleton not only controls cell morphology but also regulates cell growth, migration, differentiation and gene expression, events which are fundamental to embryogenesis, carcinogenesis and wound healing (Varedi et al, 1997). During cell adhesion different cytoskeleton components are colocalized with integrins in adhesion plaques (Meijne et al, 1994), and microfilaments are part of the plaque actin–myosin complex. In non-muscular cells actin has both a major contractile function and a cytoskeletal role. In these cells actin is attached mainly to the cytoplasmic surface of the cell membrane, either directly or through other proteins linked to certain intrinsic transmembrane proteins. Intermediate filaments enmesh the nucleus, and play a role in maintenance of nuclear-cytoplasmic pathways. Cytoskeletal proteins are involved in protein kinase C and $Ca^{2+}$/calmodulin-dependent protein kinase II signalling pathways that involve phosphorylation of intermediate filaments (Ogawara et al, 1995; Inagaki et al, 1997). Additionally, it was shown that cGMP- and cAMP-dependent protein kinases (Lamb et al, 1989; Wyatt et al, 1991) can also be involved in phosphorylation of intermediate filaments following their rearrangement. Microtubules are part of the cytoskeleton and take part in intracellular transport. The major component of microtubules is tubulin (Kreis and Vale, 1993), and this molecule also seems to be integrated in intracellular signalling and is related to intermediate filaments (Gurland and Gundersen, 1995).

Disruption of cytoskeletal proteins can change various cell functions, such as mRNA and protein expression of proteinases (Botteri et al, 1990; Young et al, 1994). Furthermore, it has been described that cycloheximide and acrylamide specifically disrupt the organization of the intermediate filament system in CV-1 kidney cells with no discernable effects on microtubules or microfilaments (Sharpe et al, 1980). The presence of intermediate filaments coincided with loss of anchorage dependency in several head and neck squamous cell carcinoma cell lines (Tomson et al, 1996). Integrins function as integrative elements in cytoskeletal arrangements and are involved in signalling events (Yamada and Miyamoto, 1995). Disruption of several cytoskeletal proteins, including actin and intermediate filaments, interferes with integrin-mediated phosphorylation of intracellular proteins (Tang et al, 1993). Furthermore, cytoskeletal arrangements and integrin surface expression seem to be related. Hendrix et al (1996, 1997) described morphological and biological changes, such as tumorigenicity, invasiveness and integrin expression of different carcinoma cell lines, that were associated with direct linkages between intermediate filaments. The remodelling of matrigel, a model ECM matrix, by tumour cells was associated with $\alpha_6\beta_4$ integrin expression in several cell lines and can be blocked by cytochalasin (Rabinovitz and Mercurio, 1996). Phosphorylation of intermediate filaments and other cytofilaments appears to be involved in cytoskeletal rearrangements, and integrins may trigger these events.

In summary, our data support the notion that microfilaments and intermediate filaments play a profound role in colorectal carcinoma cell adhesion stabilization and signal transduction into the cell during cell adhesion. As shown for B16 melanoma cells (Chopra et al, 1991), actin and intermediate filaments appear to be also involved in adhesion of tumour cells to ECM components, whereas tubulin disruption had no effect on cell adhesion. Regulation of adhesion-mediated signals occurs at various levels, and cytoskeleton interactions and rearrangement of cytoskeletal components appear to play roles in these complex processes.

### REFERENCES

Agrez MV and Bates RC (1994) Colorectal cancer and the integrin family of cell adhesion receptors: Current status and future directions. *Eur J Cancer* **14**: 2166–2170

Akiyama SK, Yamada SS, Yamada KM and Laflamme SE (1994) Transmembrane signal transduction by integrin cytoplasmic domains expressed in single-subunit chimeras. *J Biol Chem* **269**: 15961–15964

Ben-Ze'ev A (1997) Cytoskeletal and adhesion proteins as tumor suppressors. *Curr Opin Cell Biol* **9**: 99–108

Ben-Ze'ev A and Raz A (1985) Relationship between the organization and synthesis of vimentin and the metastatic capability of B16 melanoma cells. *Cancer Res* **45**: 2632–2641

Botteri FM, Ballmer-Hofer K, Rajput B and Nagamine Y (1990) Disruption of cytoskeletal structures results in the induction of the urokinase-type plasminogen activator gene expression. *J Biol Chem* **265**: 13327–13334

 © 1999 Cancer Research Campaign

Boyd D, Florent G, Childress-Fields K and Brattain MG (1988) Alteration in the behavior of a colon carcinoma cell line by extracellular matrix components. *Cancer Lett* **41**: 81–90

Burtin P, Chavanel G and Foidart JM (1983) Immunofluorescence study of the antigens of the basement membrane and the peritumoral stroma in human colonic adenocarcinomas. *Ann NY Acad Sci* 229–236

Chen YP, O'Toole TE, Shipley T, Forsyth J, Laflamme SE, Yamada KM, Shattil SJ and Ginsberg MH (1994) 'Inside-out' signal transduction inhibited by isolated integrin cytoplasmic domains. *J Biol Chem* **269**: 18307–18310

Chopra H, Hatfield JS, Chang YS, Grossi IM, Fitzgerald LA, O'Gara CY, Marnett LJ, Diglio CA, Taylor JD and Honn KV (1988) Role of tumor cytoskeleton and membrane glycoprotein IRGpIIb/IIIa in platelet adhesion to tumor cell membrane and tumor cell-induced platelet aggregation. *Cancer Res* **48**: 3787–3800

Chopra H, Fligiel SE, Hatfield JS, Nelson KK, Diglio CA, Taylor JD and Honn KV (1990) An in vivo study of the role of the tumor cell cytoskeleton in tumor cell-platelet-endothelial cell interactions. *Cancer Res* **50**: 7686–7696

Chopra H, Timar J, Chen YQ, Rong XH, Grossi IM, Fitzgerald LA, Taylor JD and Honn KV (1991) The lipoxygenase metabolite 12(S)-HETE induces a cytoskeleton-dependent increase in surface expression of integrin $\alpha_{IIb}\beta_3$ on melanoma cells. *Int J Cancer* **49**: 774–786

Danecker GW, Piazza AJ, Stelle GD and Mercurio AM (1989) Relationship between extracellular matrix interactions and degree of differentiation in human colon carcinoma cell lines. *Cancer Res* **49**: 681–686

Doerr R, Zvibel I, Chiuten D, D'Olimpio J and Reid LM (1989) Clonal growth of tumors on tissue-specific biomatrices and correlation with organ site specificity of metastasis. *Cancer Res* **49**: 384–339

Gong J, Wang DH, Sun LZ, Zborowska E, Willson JKV and Brattain MG (1997) Role of α5β1 integrin in determining malignant properties of colon carcinoma cells. *Cell Growth Diff* **8**: 83–90

Grigioni WF, Biagini G, Errico AD, Milani M, Villanacci V, Garbisa S, Mattioli S, Gozzetti G and Mancini AM (1986) Behaviour of basement membrane antigens in gastric and colorectal cancer: Immunohistochemical study. *Acta Pathol Jpn* **36**: 173–184

Guan JL, Trevithick JE and Hynes RO (1991) Fibronectin/integrin interaction induces tyrosine phosphorylation of a 120 kDa protein. *Cell Regul* **2**: 951–964

Gumbiner BM (1996) Cell adhesion: The molecular basis of tissue architecture and morphogenesis. *Cell* **84**: 345–357

Gurland G and Gundersen GG (1995) Stable, detyrosinated microtubules function to localize vimentin intermediate filaments in fibroblasts. *J Cell Biol* **131**: 1275–1290

Haier J, Nasralla M, Buhr HJ and Nicolson GL (1998) Differential integrin-associated adhesion to extracellular matrix in colon cancer cells with low metastatic potential or highly metastatic potential to the liver. *Langenbecks Arch Chir Suppl* 307–313

Hendrix MJC, Seftor EA, Chu YW, Trevor KT and Seftor REB (1996) Role of intermediate filaments in migration, invasion and metastasis. *Cancer Metast Rev* **15**: 507–525

Hendrix MJ, Seftor EA, Seftor RE and Trevor KT (1997) Experimental co-expression of vimentin and keratin intermediate filaments in human breast cancer cells results in phenotypic interconversion and increased invasive behavior. *Am J Pathol* **150**: 483–495

Herzberg F, Schöning M, Schirner M, Topp M, Thiel E and Kreuser ED (1996) IL-4 and TNF-α induce changes in integrin expression and adhesive properties and decrease the lung-colonizing potential of HT-29 colon carcinoma cells. *Clin Exp Metastasis* **14**: 165–175

Horwitz A, Duggan K, Buck C, Beckerle MC, Burrigde K (1986) Interaction of plasma membrane fibronectin receptor with talin: a transmembrane linkage. *Nature* **320**: 531–533

Inagaki N, Goto H, Ogawara M, Nishi Y, Ando S and Inagaki M (1997) Spatial patterns of $Ca^{2+}$ signals define intracellular distribution of a signaling by $Ca^{2+}$/calmodulin-dependent protein kinase II. *J Biol Chem* **272**: 25195–25199

Inufusa H, Nakamura M, Adachi T, Nakatani Y, Shindo K, Yasutomi M and Matsuura H (1995) Localization of oncofetal and normal fibronectin in colorectal cancer. *Cancer* **75**: 2802–2808

Jewell K, Kapron-Bras C, Jeevaratnam P and Dedhar S (1995) Stimulation of tyrosine phosphorylation of distinct proteins in response to antibody-mediated ligation and clustering of α3 and α6 integrins. *J Cell Sci* **108**: 1165–1174

Kemperman H, Wijnands YM and Roos E (1997) Alpha V integrins on HT-29 colon carcinoma cells: adhesion to fibronectin is mediated solely by small amounts of alphaVbeta6, and alphaVbeta5 is codistributed with actin fibers. *Exp Cell Res* **234**: 156–164

Kim WH, Jun SH, Kibbey MC, Thompson EW and Kleinman HK (1994) Expression of beta 1 integrin in laminin-adhesion-selected human colon cancer cell lines of varying tumorigenicity. *Invasion Metastasis* **14**: 147–155

Kornberg LJ, Earp HS, Turner CE, Prockop C and Juliano RL (1991) Signal transduction by integrins: Increased protein tyrosine phosphorylation caused by clustering of β1 integrins. *Proc Natl Acad Sci USA* **88**: 8392–8396

Kreis T and Vale R (1993) Guidebook of the cytoskeletal and motor proteins. Oxford University Press, Oxford

Lamb NJ, Fernandez A, Feramisco JR and Welch WJ (1989) Modulation of vimentin containing intermediate filament distribution and phosphorylation in living fibroblasts by the cAMP-dependent protein kinase. *J Cell Biol* **108**: 2409–2422

Lehmann M, Rabenandrasana C, Tamura R, Lissitzky JC, Quaranta JP and Marvaldi J (1994) A monoclonal antibody inhibits adhesion to fibronectin and vitronectin of a colon carcinoma cell line and recognizes the integrins αvβ3, αvβ5 and αvβ6. *Cancer Res* **54**: 2102–2107

Lindmark G, Gerdin B, Pahlman L, Glimelius B, Gehlsen K and Rubin K (1993) Interconnection of integrins alpha 2 and alpha 3 and structure of the basal membrane in colorectal cancer: relation to survival. *Eur J Surg Oncol* **19**: 50–60

Mainiero F, Pepe A, Wary KK, Spinardi L, Mohammadi M, Schlessinger J and Giancotti FG (1995) Signal transduction by the α6β4 integrin: Distinct β4 subunit sites mediate recruitment of Shc/Grb2 and association with cytoskeleton of hemidesmosomes. *EMBO* **14**: 4470–4481

Meijne AM, Casey DM, Feltkamp CA and Roos E (1994) Immuno-EM localization of the beta 1 integrin subunit in wet-cleaved fibronectin-adherent fibroblasts. *J Cell Sci* **107**: 1229–1239

Miyamoto S, Akiyama SK and Yamada KM (1995) Synergistic roles for receptor occupancy and aggregation in integrin transmembrane function. *Science* **267**: 883–885

Mooney DJ, Langer R and Ingber DE (1995) Cytoskeletal filament assembly and the control of cell spreading and function by extracellular matrix. *J Cell Sci* **108**: 2311–2320

Nicolson GL (1988*a*) Cancer metastasis: tumor cell and host properties important in colonization of specific secondary sites. *Biochim Biophys Acta* **948**: 175–224

Nicolson GL (1998*b*) Organ specificity of tumor metastasis: role of preferential adhesion, invasion and growth of malignant cells at specific secondary sites. *Cancer Metastasis Rev* **7**: 143–188

Nicolson GL (1989) Metastatic tumor cell interactions with endothelium, basement membrane and tissue. *Curr Op Cell Biol* **1**: 1009–1019

Nicolson GL (1991) Tumor and host molecules important in the organ preference of metastasis. *Cancer Biol* **2**: 143–154

Nicolson GL (1995) Tumor interactions with the vascular endothelium and their role in cancer metastasis. In: *Epithelial–mesenchymal interactions in cancer.* Goldberg ID and Rosen EM (ed.) pp. 123–156. Birkhäuser Verlag: Basel

Ogawara M, Inagaki N, Tsujimura K, Takai Y, Sekimata M, Ha MH, Imajoh Ohmi S, Hirai S, Ohno S and Sugiura H (1995) Differential targeting of protein kinase C and CaM kinase II signaling to vimentin. *J Cell Biol* **131**: 1055–1066

Orian-Rousseau V, Aberdam D, Rousselle P, Messent A, Gavrilovic J, Meneguzzi G, Kedinger M and Simon-Assmann P (1998) Human colonic cancer cells synthesize and adhere to laminin-5. Their adhesion to laminin-5 involves multiple receptors among which is integrin α2β1. *J Cell Sci* **111**: 1993–2004

Price JE, Daniels LM, Campbell DE and Giavazzi R (1989) Organ distribution of experimental metastasis of a human colorectal carcinoma injected in nude mice. *Clin Exp Metatasis* **7**: 55–68

Rabinovitz I and Mercurio AM (1996) The integrin α6β4 and the biology of carcinoma. *Biochem Cell Biol* **74**: 811–821

Sawada H, Wakabayashi H, Nawa A, Mora E, Cavanaugh PG and Nicolson GL (1996) Differential motility stimulation but not growth stimulation or adhesion of metastatic human colorectal carcinoma cells by target organ-derived liver sinusoidal endothelial cells. *Clin Exp Metastasis* **14**: 308–314

Schreiner C, Bauer J, Margolis M and Juliano RL (1991) Expression and role of integrins in adhesion of human colonic carcinoma cells to extracellular matrix components. *Clin Exp Metastasis* **9**: 163–178

Schwarz MA, Schaller MD and Ginsberg MH (1995) Integrins: Emerging paradigms of signal transduction. *Annu Rev Cell Dev Biol* **11**: 549–599

Seftor REB, Seftor EA, Gehlsen KR, Steler-Stevenson WG, Brown PD, Ruoslathi E and Hendrix MJC (1992) Role of the αvβ3 integrin in human melanoma cell invasion. *Proc Natl Acad Sci USA* **89**: 1557–1561

Seufferlein T and Rozengurt E (1994) Lysophosphatidic acid stimulates tyrosine phosphorylation of focal adhesion kinase, paxillin and p130. *J Biol Chem* **269**: 9345–9351

Sharpe AH, Chen LB, Murphy JR and Fields BN (1980) Specific disruption of vimentin filament organization in monkey kidney CV-1 cells by diphtheria toxin, exotoxin A, and cycloheximide. *Proc Natl Acad Sci USA* **77**: 7267–7271

© 1999 Cancer Research Campaign

Stallmach A, Von Lampe B, Matthes H, Bornhöft G and Riecken EO (1992) Diminished expression of integrin adhesion molecules on human colonic epithelial cells during the benign to malignant transformation. *Gut* **33**: 342–346

Streit M, Schmidt R, Hilgenfeld RU, Thiel E and Kreuser ED (1996) Adhesion receptors in malignant transformation and dissemination of gastrointestinal tumors. *Recent Results Cancer Res* **142**: 19–50

Takazawa H (1995) Association between expression of integrin (VLA-3, VLA-5) and malignancy in human colon-cancer. *Nippon Rinsho* **53**: 1672–1677

Tang DG, Timar J, Grossi IM, Renaud C, Kimler VA, Diglio CA, Taylor JD and Honn KV (1993) The lipoxygenase metabolite 12(S)-HETE induces a protein kinase C-dependent cytoskeletal rearrangement and retraction of microvascular endothelial cells. *Exp Cell Res* **207**: 361–375

Tang DG, Diglio CA and Honn KV (1994) Activation of microvascular endothelium by eicosanoid 12(S)-hydroxyeicosatetraenoic acid leads to enhanced tumor cell adhesion via up-regulation of surface expression of $\alpha v\beta 3$ integrin: A posttranscriptional, protein kinase C- and cytoskeleton-dependent process. *Cancer Res* **54**: 1119–1129

Tomson AM, Scholma J, Meijer B, Koning JG, De Jong KM and Van Der Werf M (1996) Adhesion properties, intermediate filaments and malignant behavior of head and neck squamous cell carcinoma cells in vitro. *Clin Exp Metastasis* **14**: 501–511

Varedi M, Ghahary A, Scott PG and Tredget EE (1997) Cytoskeleton regulates expression of genes for transforming growth factor-beta 1 and extracellular matrix proteins in dermal fibroblasts. *J Cell Physiol* **172**: 192–199

Wyatt TA, Lincoln TM and Pryzwansky KB (1991) Vimentin is transiently co-localized with and phosphorylated by cyclic GMP-dependent protein kinase in formyl-peptide-stimulated neutrophils. *J Biol Chem* **266**: 21274–21280

Yamada KM and Geiger B (1997) Molecular interactions in cell adhesion complexes. *Curr Op Cell Biol* **9**: 76–85

Yamada KM and Miyamoto S (1995) Integrin transmembrane signaling and cytoskeletal control. *Curr Op Cell Biol* **7**: 681–689

Yatohgo T, Izumi M, Kashiwagi H and Hayashi M (1988) Novel purification of vitronectin from human plasma by heparin affinity chromatography. *Cell Struc Funct* **13**: 281–292

Young MR, Charboneau S, Lozano Y, Djordjevic A and Young ME (1994) Activation of the protein kinase a signal transduction pathway by granulocyte-macrophage colony-stimulating factor or by genetic manipulation reduces cytoskeletal organization in Lewis lung carcinoma variants. *Int J Cancer* **56**: 446–451

 *© 1999 Cancer Research Campaign*
